# Supplementary material for: Replication Variance of African and Asian Lineage Zika Virus Strains in Different Cell Lines, Mosquitoes and Mice
Source: Microorganisms. 2021 Jun 9;9(6):1250. doi: 10.3390/microorganisms9061250 (PMC8230095; doi:10.3390/microorganisms9061250)
Supplement: Supplementary file 1 [file microorganisms-09-01250-s001.zip › microorganisms-1207399-supplementary.pdf]

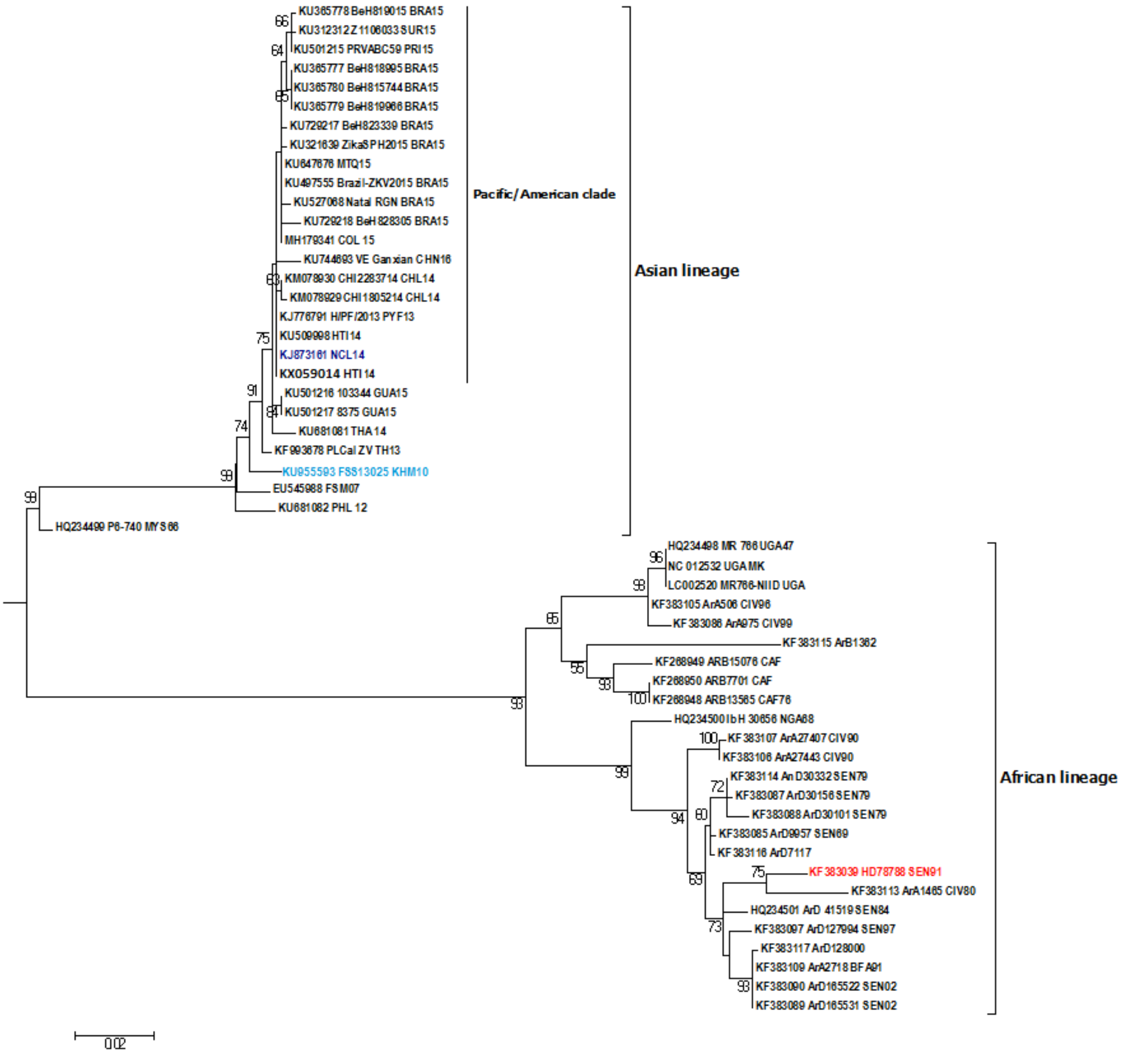

Supplementary Figure S1: Phylogenetic tree of ZIKV strains based on partial nonstructural protein 5 gene sequences

Multiple sequence alignment of ZIKV strains representing both African and Asian lineage available in Genbank. ZIKV strains used in this study: Cambodia isolate FSS13025 (light blue), New Caledonian isolate (dark blue) both belonging to the Asian lineage, and African isolate HD78788 (red). MEGA6 software was used to perform phylogenetic analyses by applying maximum-likelihood method and the general time reversible model with 1,000 bootstrap resampling. Spondweni virus (GenBank: AF013406) was used to root the tree. Scale bar indicates nucleotide substitutions per site.

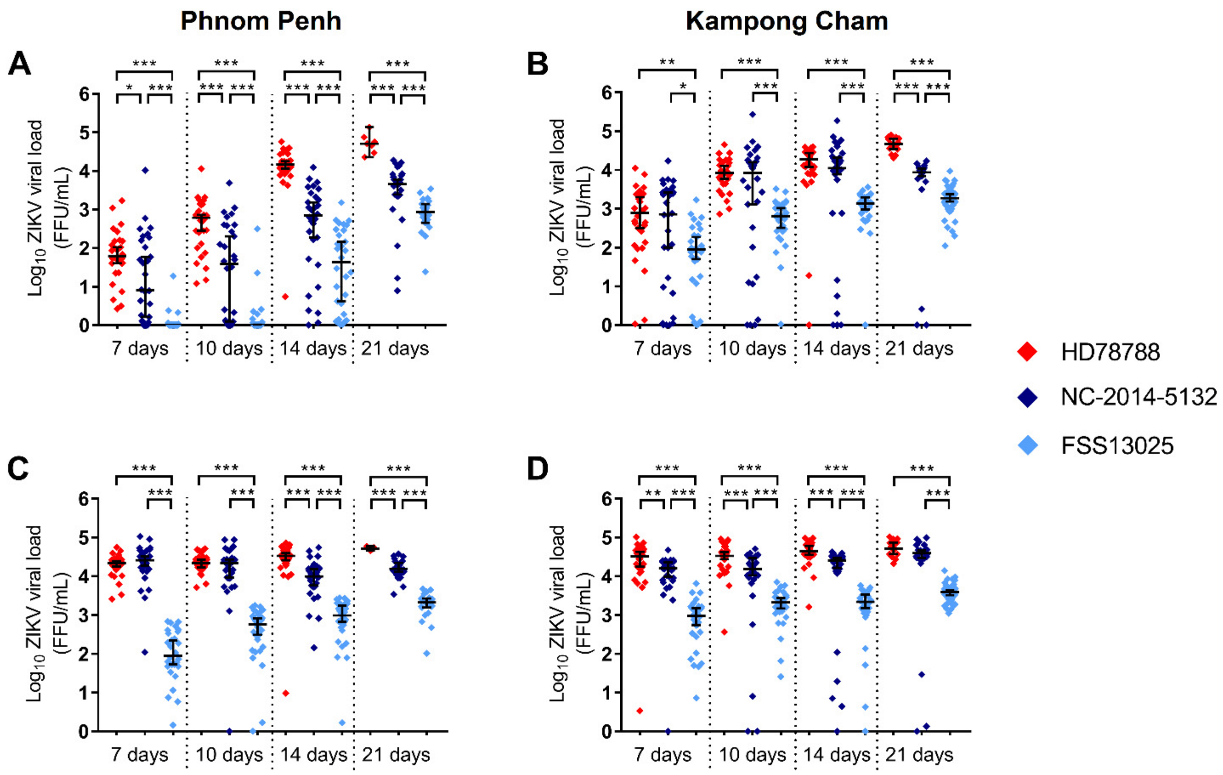

Supplementary Figure S2: Viral load of two different Cambodian *Ae. aegypti* mosquito populations.

Cambodian *Ae. aegypti* populations from urban Phnom Penh (A, C) and rural Kampong Cham (B, D) were infected with ZIKV strain HD7888 (red), NC-2014-5132 (dark blue) or FSS13025 (light blue) by infectious blood meal. Virus titers in head, legs and wings (A, B) as well as bodies (C, D) were determined by RT-qPCR and individual results are plotted with median and 95 % CI. Statistical differences determined with Fisher's exact test are marked by asterisks: * p<0.05, ** p<0.01, *** p<0.001.

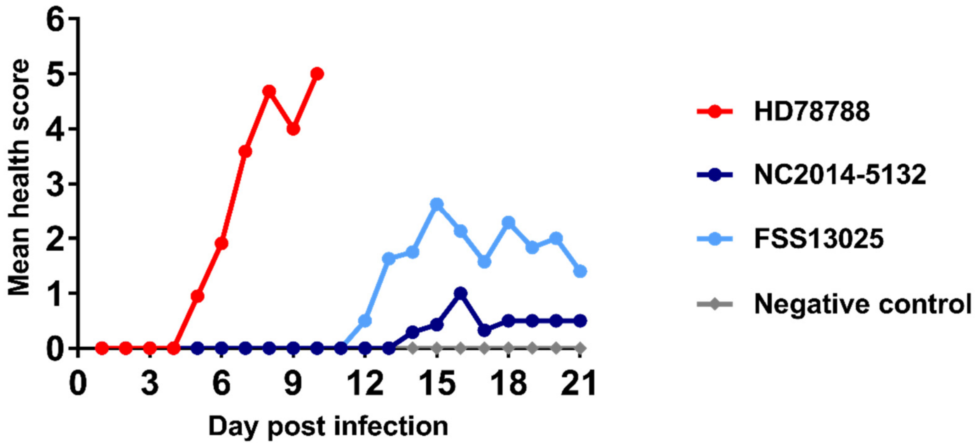

Supplementary Figure S3: Health score in neonatal Swiss mice after ZIKV infection

One-day old Swiss mice infected with ZIKV strain HD7888 (red), NC-2014-5132 (dark blue) or FSS13025 (light blue) by intracerebral injection. Over 21 days or until death the clinical symptoms were scored as follows: 0 healthy, 1 fever, 2 weaker and more emaciated, 3 limb weakness and back pain, 4 hind-limb or fore-limb paralysis and tremors, and 5 death.

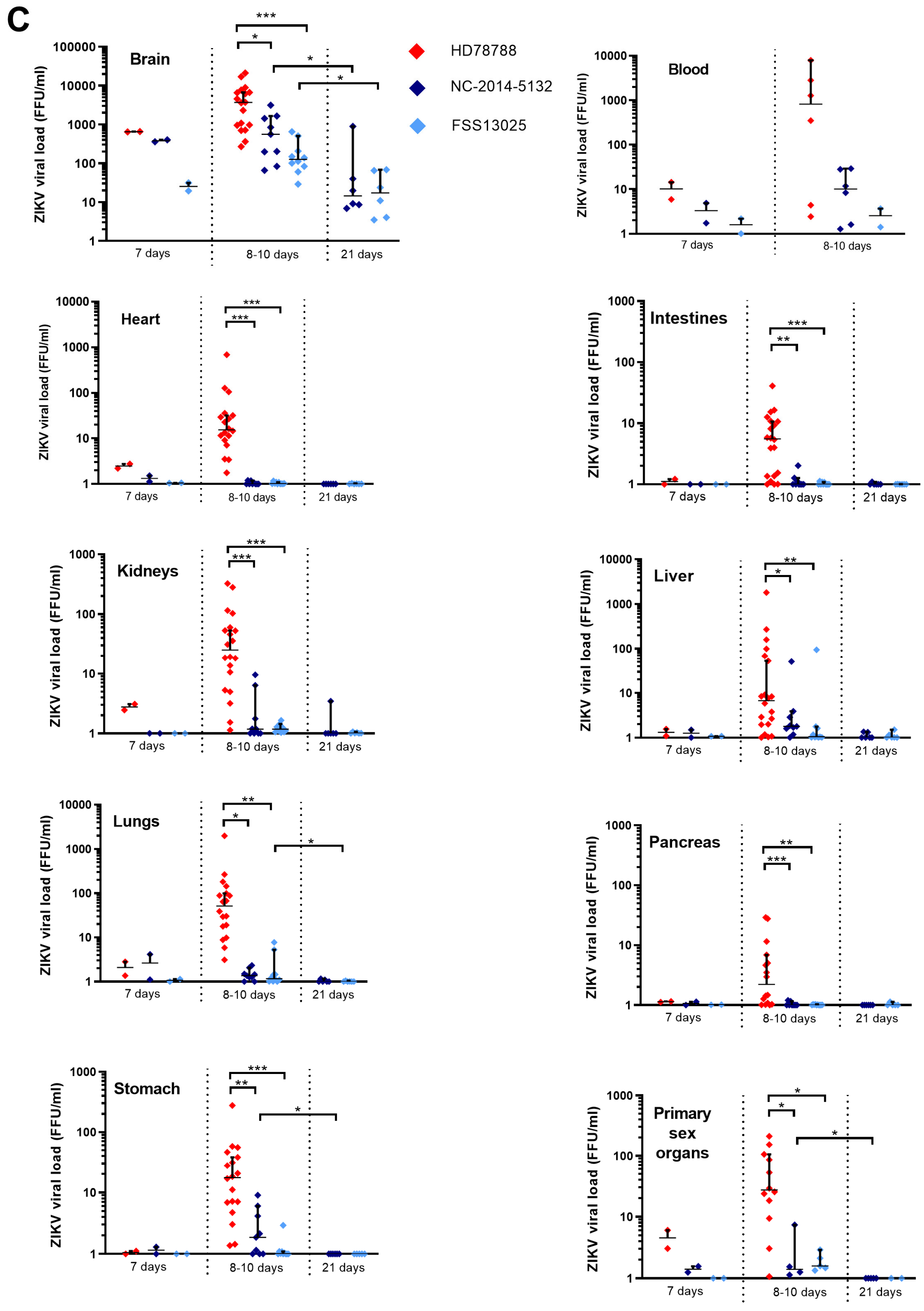
C
Brain
Blood
Heart
Intestines
Kidneys
Liver
Lungs
Pancreas
Stomach
Primary sex organs
HD78788
NC-2014-5132
FSS13025
ZIKV viral load (FFU/ml)
7 days
8-10 days
21 days

Supplementary Figure S4: Viral load ZIKV in different organs of neonatal Swiss mice

After death at day 7, 8 and 10 p.i. or euthanasia at day 7, 8-10 and 21 p.i., the viral load in different organs was determined by RT-qPCR. Median viral titers with 95% CI for blood and organs are shown. Statistical differences determined with Kruskal-Wallis test (Dunn's multiple comparison correction) are marked by asterisks: * $p<0.05$, ** $p<0.01$, *** $p<0.001$.

Supplementary material

Supplementary Table S1: Infection, dissemination and transmission rates of Phnom Penh *Aedes aegypti* mosquito population

Days after infection	HD78788 (4.1 x 10^6 FFU/mL)$^{\Phi}$	NC-2014-5132 (4.7 x 10^6 FFU/mL)$^{\Phi}$	FSS13025 (4.6 x 10^6 FFU/mL)$^{\Phi}$
	**Infection rate ***		
**7**	100% (30/30)	100% (30/30)	100% (30/30)
**10**	100% (30/30)	96.7% (29/30)	96.7% (29/30)
**14**	100% (30/30)	100% (30/30)	100% (30/30)
**21**	100% (7/7)	100% (30/30)	100% (22/22)
	**Dissemination rate #**		
**7**	100% (30/30)	80.0% (24/30)	26.7% (8/30)
**10**	100% (30/30)	82.8% (24/29)	55.2% (16/29)
**14**	100% (30/30)	96.7% (29/30)	100% (30/30)
**21**	100% (7/7)	100% (30/30)	100% (22/22)
	**Transmission rate §**		
**7**	6.7% (2/30)	4.2% (1/24)	0% (0/8)
**10**	50.0% (15/30)	4.2% (1/24)	6.3% (1/16)
**14**	73.3% (22/30)	17.2% (5/29)	10.0% (10/30)
**21**	100% (7/7)	33.3% (10/30)	40.9% (9/20)

$^{\Phi}$ Viral blood meal titer

* Number of mosquitoes with ZIKV-positive body/total number of blood-fed mosquitoes

Number of mosquitoes with ZIKV-positive head, legs, wings/number of mosquitoes with ZIKV-positive body

§ Number of mosquitoes with ZIKV-positive saliva/number of mosquitoes with ZIKV-positive heads, legs, wings

Supplementary Table S2: Infection, dissemination and transmission rates of Kampong Cham *Aedes aegypti* mosquito population

Days after infection	HD78788 (1.5 x 10^6 FFU/mL)$^{\Phi}$	NC-2014-5132 (4.8 x 10^5 FFU/mL)$^{\Phi}$	FSS13025 (1.2 x 10^6 FFU/mL)$^{\Phi}$
	**Infection rate ***		
**7**	100% (30/30)	96.7% (29/30)	100% (30/30)
**10**	100.% (30/30)	93.3% (28/30)	100% (30/30)
**14**	100% (30/30)	96.7% (29/30)	96.7% (29/30)
**21**	100% (15/15)	95.7% (22/23)	100% (50/50)
	**Dissemination rate #**		
**7**	100% (30/30)	93.1% (27/29)	100% (30/30)
**10**	100% (30/30)	96.4% (27/28)	100% (30/30)
**14**	96.7% (29/30)	93.1% (27/28)	100% (29/29)
**21**	100% (15/15)	100% (22/22)	100% (50/50)
	**Transmission rate §**		
**7**	36.7% (11/30)	3.7% (1/27)	13.3% (4/30)
**10**	80.0% (24/30)	18.5% (5/27)	36.7% (11/30)
**14**	96.6% (28/29)	33.3% (9/27)	82.8% (24/29)
**21**	86.7% (13/15)	68.2% (15/22)	78.0% (39/50)

$^{\Phi}$ Viral blood meal titer

* Number of mosquitoes with ZIKV-positive body/total number of blood-fed mosquitoes

Number of mosquitoes with ZIKV-positive head, legs, wings/number of mosquitoes with ZIKV-positive body

§ Number of mosquitoes with ZIKV-positive saliva/number of mosquitoes with ZIKV-positive heads, legs, wings

Supplementary Table S3: Survival rates of ZIKV infected mosquitoes

Mosquito population	ZIKV strain	No. of engorged females (day 0 p.i.)	No. of females removed for analysis (before day 21 p.i.)	No. of alive females day 21 p.i.	Survival rate
Phnom Penh	HD78788	199	90	7	6.4%
	NC-2014-5132	256	90	30	18.1%
	FSS13025	242	90	22	14.5%
Kampong Cham	HD78788	224	90	15	11.2%
	NC-2014-5132	266	90	23	13.1%
	FSS13025	266	90	50	28.4%

Supplementary Table S4: Viral load in organs of neonatal Swiss mice after death or euthanasia

Organ	HD78788 (death) Median viral load* (95% CI)						NC-2014-5132 (euthanasia) Median viral load* (95% CI)						FSS13025 (euthanasia) Median viral load* (95% CI)					
	n	Day 7 p.i.	n	Day 8 p.i.	n	Day 10 p.i.	n	Day 7 p.i.	n	Day 10 p.i.	n	Day 21 p.i.	n	Day 7 p.i.	n	Day 10 p.i.	n	Day 21 p.i.
Brain	2	648 (643 – 653)	13	3730 (713 – 7720)	5	1080 (361 – 16900)	2	381 (358 – 403)	9	555 (83 – 1640)	6	14 (7 – 894)	2	25 (19 – 31)	10	126 (60 – 505)	6	17 (4 – 68)
Blood	2	10 (6 –14)	4	1575 (3 – 7910)	2	642 (5 – 1280)	2	3 (2 – 5)	6	10 (1 - 29)	0	0	2	1.6 (1 – 2.2)	2	3 (1 – 4)	0	0
Heart	2	2.5 (2.2 – 2.8)	13	16 (7 – 105)	5	11 (2 – 32)	2	1.3 (1.1 – 1.5)	9	1 (1 – 1.2)	6	1 (1 – 1)	2	1 (1 – 1.1)	10	1 (1 – 1.1)	6	1 (1 – 1.1)
Intestines	2	1.1 (1 – 1.2)	15	4 (1 – 11)	5	8 (6 – 41)	2	1 (1 – 1)	9	1 (1 – 1.3)	6	1 (1 – 1.1)	2	1 (1 – 1)	10	1 (1 – 1.1)	6	1 (1 – 1)
Kidneys	2	2.8 (2.5 – 3.1)	15	19 (5 – 53)	5	46 (11 – 115)	2	1 (1 – 1)	9	1.2 (1 – 6)	6	1 (1 – 3.5)	2	1 (1 – 1)	10	1.2 (1 – 1.4)	6	1 (1 – 1.1)
Liver	2	1.3 (1.1 – 1.6)	15	3 (1 – 8)	5	159 (68 – 1810)	2	1.3 (1 – 1.5)	9	2 (1 – 4)	6	1 (1 – 1.4)	2	1 (1 – 1.1)	10	1 (1 – 1.2)	6	1 (1 – 2)
Lung	2	2 (1 – 3)	13	39 (9 – 89)	5	99 (19 – 1980)	2	3 (1 – 4)	9	1.4 (1 – 2)	6	1 (1 – 1.2)	2	1.1 (1 – 1.2)	10	1.2 (1 – 5)	6	1 (1 – 1)
Pancreas	2	1.1 (1.1 – 1.2)	11	1.3 (1 – 28)	5	5 (1 – 7)	2	1.1 (1 – 1.2)	9	1 (1 – 1.2)	6	1 (1 – 1)	2	1 (1 – 1)	10	1 (1 – 1)	5	1 (1 – 1.2)
Stomach	2	1 (1 – 1.1)	13	7 (3 – 28)	5	38 (17 – 277)	2	1.2 (1 - 1.3)	9	2 (1 – 6)	6	1 (1 – 1)	2	1 (1 – 1)	10	1 (1 – 1.1)	5	1 (1 – 1)
Primary sex organs	2	5 (3 – 6)	9	29 (9 – 153)	3	18 (1.1 – 53)	2	1.4 (1.3 – 1.6)	4	1.4 (1.2 – 7)	5	1 (1 – 1)	2	1 (1 – 1)	5	2 (1 – 3)	2	1 (1 – 1)

* FFU/mL
